# Ecological and phylogenetic predictors of mobbing behavior in a tropical dry forest

**DOI:** 10.1002/ece3.4683

**Published:** 2018-12-12

**Authors:** Hevana Santana de Lima, Flor Maria G. Las‐Casas, Jonathan R. Ribeiro, Thiago Gonçalves‐Souza, Luciano N. Naka

**Affiliations:** ^1^ Laboratório de Ornitologia, Departamento de Zoologia, Centro de Biociências Universidade Federal de Pernambuco Recife Brazil; ^2^ Departamento de Biologia Universidade Federal Rural de Pernambuco Recife Brazil; ^3^ Department of Organismic and Evolutionary Biology Harvard University Cambridge Massachusetts

**Keywords:** dry forest, ecological interactions, functional traits, mobbing, phylogenetic structure

## Abstract

Mobbing represents a well‐known anti‐predatory behavior, where potential prey display aggressively against a predator. Despite considerable experimental and descriptive work, no models predict species participation in mobbing assemblages. Here, we aimed to understand why some bird species engage in this behavior, while others do not, and what factors can be used to predict mobbing engagement within an avian community. We investigated whether certain functional traits, such as body size, foraging guild, foraging mode, and strata, as well species abundance and evolutionary relatedness, are important mobbing predictors. To address these goals, we simulated the presence of the Ferruginous Pygmy‐Owl (*Glaucidium brasilianum*) by broadcasting its voice in 230 experiments conducted in 115 points, systematically distributed in a dry forest of northeastern Brazil. We compared these results to 162 avian surveys (point counts) conducted in the same area. Our avian surveys detected 108 bird species (local avian community), whereas our playback experiments attracted 72 species (mobbing assemblage). In general, small, canopy insectivorous or frugivorous birds dominated the mobs. The best mobbing predictors were body mass and guild, whereas species abundance, foraging mode, and strata were not retained in the best models. We found a strong phylogenetic component in body mass and mobbing propensity (almost 90% of the species and individuals participating in the mobs were passerines). At the community level, we found significant differences in the functional and phylogenetic structure of the mobbing assemblage in relation to the avian community. Our results suggest that mobbing behavior is tightly associated with predation risk and the capacity of individual species to find and detect predators, and that functional and phylogenetic features can predict species participation in this complex animal behavior.

## 1 INTRODUCTION

In the animal world, the risk of being killed triggers anti‐predatory behaviors to avoid potential predators. These behaviors may affect different aspects of animal decision‐making, such as time and place for feeding and handling food, sociality, as well as activity and vigilance patterns (Lima & Dill, 1990). Once a potential predator is detected, however, rather than escaping and hiding, some species engage in risky mobbing attacks that can put their own lives in peril. Mobbing represents a well‐known anti‐predatory behavior, where members of one or more species display aggressive behaviors aiming to disclose the presence of a predator and eventually chase it away (Hartley, 1950). Although mobbing is widespread in the animal world, studies have barely evaluated which ecological variables drive the expression of this defensive behavior against predators. Untangling those drivers is a necessary step to understand mobbing function and origin, two aspects that have been elusive to behavioral ecologists and remain poorly understood. One way of moving the field forward is by evaluating which species in a local community engage in this behavior and assess whether particular traits can predict species participation in the mobs. It also remains untested, whether close relatives share this behavior or whether is widespread throughout the community.

Several hypotheses were invoked to explain the origins of mobbing and understand why potential preys may face and fight a predator, putting their lives in peril, rather than hide or scape. Hypotheses include individual altruism, parental care, and selfish behavior (where mobbing individuals avoid predation; Ostreiher, 2003). Mobbing has been documented in several groups of vertebrates, such as fish (Bshary, Wickler, & Fricke, 2002; Dominey, 1983; Smith, 1992) and mammals (Bartecki & Heymann, 1987; De Stephanis et al., 2015; Graw & Manser, 2007; Smythe, 1970; Tamura, 1989), but it is in birds that this behavior is more widespread and possibly better understood (Altmann, 1956; Betts, Hadley, & Doran, 2005; Chandler & Rose, 1988; Curio, 1978; Doran, Gulezian, & Betts, 2005). Among birds, mobbing is usually directed at different kinds of potential predators, including reptiles (Breviglieri & Romero, 2016; Cunha & Fontenelle, 2014; Koboroff, Kaplan, & Rogers, 2013), mammals (Cunha & Fontenelle, 2014; Cunha, Specht, & Brites, 2013; Silva & Ferreira, 2013), and other birds, particularly hawks and eagles (Accipitridae), falcons and kestrels (Falconidae), and owls (Strigidae; Altmann, 1956; Cunha & Fontenelle, 2014; Motta‐Junior, 2007; Nijman, 2004; Pavey & Smyth, 1998; Pettifor, 1990).

In the Neotropics, the Ferruginous Pygmy‐Owl (*Glaucidium brasilianum*) is one of the most frequently mobbed species (Cunha & Vasconcelos, 2009; Cunha et al., 2013; Motta‐Junior, 2007). The vocalization of this small and relatively common owl results in an immediate reaction from mobbing birds (Cunha & Vasconcelos, 2009). The imitation of the vocalization of the Ferruginous Pygmy‐Owl in the field often attracts many otherwise silent or hidden birds, and represents a tool often used by ornithologists and birdwatchers alike to disclose the presence of many different bird species. In fact, mobbing on the Ferruginous Pygmy‐Owl is possibly one of the most well‐studied cases in the Neotropics (Cunha & Vasconcelos, 2009; Reudink, Nocera, & Curry, 2007; Sandoval & Wilson, 2012; Tilgar & Moks, 2015). Nearly, 250 bird species from 27 different avian families display mobbing behaviors against this species throughout its range (Data available in the Dryad Digital Repository, see Data Accessibility for more information). Despite this massive community‐wide behavioral response, predicting whether those species responding to predators share similar traits and a common evolutionary history is a new step forward to understand the origin and maintenance of mobbing behavior.

While considerable descriptive and experimental work on mobbing has already been conducted, one particular aspect of this behavior remains largely unexplored: why some species are frequent mobbers, while others never or rarely engage in such behavior? To answer this question, it is necessary to know not only which species engage in mobbing in a given community, but also which ones are present in the area and do not participate in the mobs. This approach will allow us to assess how predictable is the participation of mobbing and test whether particular functional traits can predict species participation in the mobs.

Here, we evaluate how size, trophic guild, foraging strata, foraging mode, abundance, and phylogenetic relatedness are related to mobbing participation. We hypothesize that smaller species will be more likely to participate in the mobs because they are more likely to be predated upon, although under the parental care hypothesis larger birds could have an advantage and have a better chance in protecting their offspring. We also postulate that the kind of food consumed by birds (feeding guild) and the way they look for it (foraging mode) may influence their rate of encounter with a potential predator. Foraging mode determines the birds’ general activity patterns and it may define vigilance rates (Hua & Sieving, 2016). More active feeding foragers, such as gleaners, or more observant species, such as salliers, may be more common in the mobs than other species. Canopy bird species will more likely encounter Pygmy‐Owls and may therefore dominate the mobbing assemblages. Finally, we argue that species abundance is an important variable to take into consideration. More common species may be more prone to join the mobs, simply by being close to a vocalizing owl. By testing these traits, we can build predictions on which part of the community will attend the mobs and create models of mobbing attendance irrespectively of the geographic location of the study area.

In this study, we conducted playback experiments simulating the presence of the Ferruginous Pygmy‐Owl to stimulate mobbing behavior in co‐occurring birds, in a dry forest of northeastern Brazil. Our main goals were to first characterize the assembly of birds attracted to the voice of the Pygmy‐Owl and compare this assemblage to the entire avian community found at the study site. By doing this, we aimed to understand whether some functional traits are better represented in the mobbing assemblages that it would be expected by chance alone. Finally, we evaluated whether evolutionary relatedness predicts the ability of certain birds to participate in the mobbing behavior. We can precisely test this prediction by comparing the phylogenetic patterns between mobbers and co‐occurring (non‐participant) species. This represents a novel approach to studying mobbing that aims to enlighten our understanding on an important and widespread agonistic behavior in the animal world.

## 2 MATERIAL AND METHODS

### 2.1 Study area

**Figure 1:**
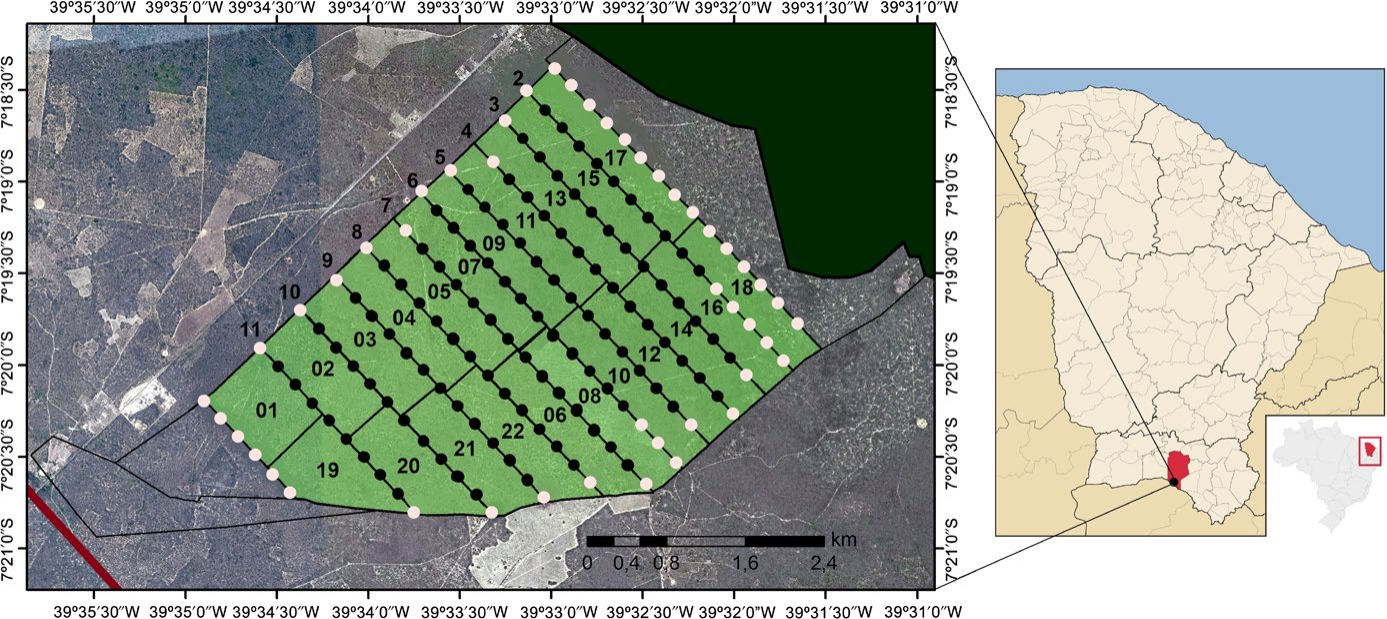
Geographic location of the Fazenda Pau D’Arco, at the Chapada do Araripe in the state of Ceará, northeastern Brazil. In detail, a map of the Fazenda and location of the 115 sampling points where we conducted playback experiments (black dots) and 162 sampling point where we conducted the acoustic census (white and black dots). Numbered lines (2 to 11) represent dirt roads that give access to management plots (1 to 22)

This study was conducted at the Fazenda Pau D’Arco, a ~2,125 ha privately owned farm, located within the Chapada do Araripe National Forest, near the municipality of Crato, in the Brazilian State of Ceará (7°18′S, 39°33′W; Figure 1). Our study site is located at the flat top of the Chapada do Araripe. The vegetation at the top of the Chapada (table top) is different from the shrubby vegetation in the surrounding Caatinga dry forest lowlands. The vegetation type on higher plateaus is locally known as *Carrasco* and includes plant elements from both the Caatinga dry forest and the Cerrado (Brazilian savanna), with small sub‐arboreal and arboreal species, and largely dominated by woody plants (Araújo, Martins, & Shepherd, 1999; Giulietti et al., 2004). The study area is being used under a legal management program that includes areas for logging. The effect of logging on the mobbing experiments will be dealt elsewhere. In the current study, we consider every point sampled as a replicate encompassing the entire variation found in the area (both natural and human‐induced).

### 2.2 Mobbing experiments

Mobbing experiments were conducted in 115 sampling points, systematically distributed every 250 m along 10 different dirt roads with lengths ranging between 4 and 5 km, which cover a total of 1,670 ha (Figure 1). The distance of 250 m between sampling points was established to ensure the independence of the data (playback broadcast was inaudible beyond 100 m), avoiding individuals to respond to successive playback experiments along the road. At each sampling point, we conducted playback experiments simulating the presence of a Ferruginous Pygmy‐Owl for 5 min. We considered as participants in the mobbing, birds that after the beginning of the playback stimulus, began to vocalize and/or approach the vocalization source, exhibiting signs of agitation (raising feathers, tail swings), producing alarm calls to other species, or moving quickly between nearby perches and visually searching for the source of the acoustic signal. We noted the number of individuals, species, and sex and age, whenever possible.

We used a recording of the natural vocalization of the Ferruginous Pygmy‐Owl obtained from the Sound Guide of the Birds of Brazil (Vielliard, 1995). The recording was edited using the software Audacity 2.0.4 (Mazzoni & Dannenberg, 2000) to remove background noises (such as background mobbing calls) that could affect the experiments. We broadcasted the vocalization using an amplifier loudspeaker Bluetooth Bright® (model 0374), connected to an iPod player, remotely controlled. The loudspeaker was hidden in the vegetation at ~2 m above the ground. All points were sampled by two observers (HSL and FMGLC), who remained silent at ~3 m from the loudspeaker, a distance that we considered adequate to visualize and listen to the birds responding to the sound stimulus, interfering minimally in their response to our own pilot tests.

All experiments were conducted at the onset of the dry season, between March 24 and April 2, 2015, end of the breeding season. At each sampling point, we conducted a morning (dawn to ~9:30) and an afternoon (from 15:30 to dusk) 5‐min playback experiment aiming to cover both peaks of avian activity along the day. Road sampling was chosen at random, and within the road, sampling points were sampled consecutively from NW to SE. We avoided sampling the same road more than once in the same day.

### 2.3 Avian surveys

The community‐wide quantitative abundance data from the study area was obtained from an independent sampling of the Fazenda’s avifauna carried out a few months before, between 15–25 October and 11–20 December 2014. Sampling was conducted by JRR and FMGLC. Point counts and mobbing experiments were conducted at the same sites, but we had 53 more point counts than experiments, totaling 162 sampling points (Figure 1). Point counts lasted for 5 min, when all birds seen or heard were recorded. Although community sampling was conducted a few months before the mobbing experiments, it did not involve any major seasonal shift in terms of migratory species or activity patterns, and we certainly did not notice any consistent temporal variation in the Fazenda’s avifauna.

### 2.4 Functional traits

To determine whether some functional traits of the mobbing bird assemblage can predict the response of species to the voice of the Pygmy‐Owl, we characterized the avifauna in terms of (a) body mass, (b) trophic guild, (c) foraging strata, and (d) foraging mode. These traits were determined for all species recorded in the study area during our community point counts, including those species that were not attracted to our playback experiments.

Avian body masses were obtained mainly from bird specimens held at the Ornithological Collection of the Universidade Federal de Pernambuco (UFPE), complemented by data from the literature (del Hoyo, Elliott, Sargatal, Christie, & deJuana, 2017) for those species not available at the collection. For each species, we used the mean weigh value of up to 10 specimens selected with no a priori reason from the collection. When fewer specimens were available, we used all available individuals to obtain mean mass values.

Trophic guilds were established based on two sources: an extensive literature search (del Hoyo et al., 2017), complemented by stomach contents available at the collection. Birds were separated in seven guilds, which included: (a) insectivores (species that feed mainly on arthropods); (b) frugivores (species that feed mainly on fruits); (c) insectivore/frugivores (species that are mainly insectivorous, but complement their diet with fruits); (d) granivores (seed eaters); (e) nectarivores (species that feed mainly on nectar); (f) omnivores (species that feed on both plant and animal material, in somewhat similar proportions); and (g) carnivores (species that feed predominantly on other vertebrates).

Foraging strata and foraging mode were classified based on our own field observations at the study area, complemented when necessary from the literature (del Hoyo et al., 2017). Species were divided into four foraging strata, including (a) ground (species that live and feed predominantly on the ground); (b) understory (species that use bushes or the lower parts of trees, up to ~2.5 m); (c) canopy (species that live predominantly on the upper part of trees, reminding that the canopy at our study site is rather low, rarely above 10 m); and (d) open air (species that forage flying above the trees). In terms of foraging mode, species were classified in 11 categories, adapted with modifications from Remsen and Robinson (1990), including (a) scavengers (species that feed on dead animals); (b) bark probers (species that search for food on trees trunks); (c) drillers (species that open holes in trunks for food); (d) flower probers (species that visit flowers); (e) fruit perchers (species that actively peak fruits perching on them); (f) gleaners (species that hunt arthropods along branches); (g) seed predators (species that feed on seeds); (h) ground probers (species that seek for food on the ground); (i) hunters (species that actively seek for moving preys); (j) salliers (species that use a sit and wait strategy to capture their prey on the wing); and (k) aerial (species that feed on the air).

### 2.5 Data analyses

#### 2.5.1 Mobbing propensity

Based on the frequency of each species at our mobbing sampling points, which was defined as the proportion of playback experiments when a given species was detected. We have established mobbing propensity to each species that participates of mobbing behavior, and this variable was used as a dependent or response variable in the functional and phylogenetic analyses.

#### 2.5.2 Functional structure

We adapted the method proposed by Duarte, Debastiani, Freitas, and Pillar (2016) to compare whether those species participating in the mobs differ in terms of their functional structure from the avian community at the study site. To do this, we compared the results from our mobbing experiments with the avian surveys (point counts). To simplify, we called this analysis Principal Coordinates of Functional Structure (PCFS), where we replaced phylogenetic dissimilarity by distances in a functional dendrogram (L. Duarte, personal communication). To define the functional distance among species, we used the generalization of Gower’s coefficient of similarity proposed by Pavoine, Vallet, Dufour, Gachet, and Daniel (2009), because it allows the inclusion of mixed variables (continuous and categorical). Similarly, we used a Principal Coordinate Analysis (PCoA) over Bray–Curtis (Krebs, 1999) dissimilarity matrices to compare species composition among predictor variables (e.g., mobbing and census).

We combined the species occurrences matrix (i.e., abundance of each species on each point of mobbing or census) with the species trait matrix to obtain a matrix where each sampled point is described by the proportion of each functional trait. Thus, we can count the frequency of each trait within guild, foraging mode, and strata. Then, we used an effect size statistic to compare how much each trait varies between mobbing and census. This comparison was made with Hedges’ *d* statistics, that is the difference between the mean of both groups, expressed as the number of standard deviations that they differ (Koricheva et al., 2013). We selected the *d* statistics because it is not affected by unequal variances (Koricheva et al., 2013). Although there are other methods for comparing group means, the *d* statistics performs well (with some bias corrected: see discussion in Koricheva et al., 2013) and was used only to help us tease apart the results of a Permutational Multivariate Analysis of Variance (PERMANOVA, McArdle & Anderson, 2001; Duarte et al., 2016) on functional and phylogenetic dissimilarities.

#### 2.5.3 Functional dissimilarities: mobbing vs. censuses

We repeated the PERMANOVA over distance matrices (obtained from the PCFS) on different subsets of trait data (see Pillar, 1999). First, we run a global model including all traits to calculate Gower’s distance. Then, we created four trait subsets by removing each trait (body mass, guild, foraging mode, and foraging strata). We recalculated Gower’s distance of each trait subset (for example, trait distance without considering species body mass), re‐run the PCFS, and compared the functional structure between mobbing and census species. By doing this, we obtained five *R* ^2^ values (from the PERMANOVA model) representing the global model and the four removed traits. Finally, we compared the effect of each trait removal with the global *R* ^2^. If a trait contributes to functional dissimilarities between mobbing and census, the *R* ^2^ value will decrease in comparison to the global model. Conversely, the *R* ^2^ of the subset with traits too similar between bird communities will increase.

#### 2.5.4 Avian phylogeny

To verify if the species’ propensity to participate in the mobs is influenced by the phylogenetic structure of the community, or in other words, whether phylogenetically closer species are more likely to behave similarly in terms of mobbing participation, we constructed a phylogenetic tree including all bird species recorded in the study area. The tree was constructed from 1,000 phylogenetic trees obtained using the Phylogeny Subset tool from the Global Phylogeny of Birds website (https://birdtree.org), which builds trees combining relaxed clock molecular trees of well‐supported avian clades with a fossil calibrated backbone with representatives from each clade (Jetz, Thomas, Joy, Hartmann, & Mooers, 2012). The 1,000 trees were imported into the software Mesquite 3.21 (Maddison & Maddison, 2018) where a *Majority‐Rule Consensus Tree* was built.

We organized the phylogenetic analysis in species and community levels (see below). We investigated in the species level whether closely related species share similar traits and how those traits influence mobbing propensity. Conversely, at the community level, we evaluated whether clade (or trait) distribution varies across mobbing and non‐mobbing species. By doing this, at this scale, we will be able to understand which clades (or traits) are likely to participate in mobbing behavior.

#### 2.5.5 Phylogenetic signal and drivers of mobbing propensity

We investigated whether there is phylogenetic signal in mobbing propensity and body size using Pagel’s lambda (Blomberg, Garland, & Ives, 2003). We also evaluated whether different bird traits (body size, abundance, guild, foraging mode, and foraging strata) affect mobbing propensity with a Phylogenetic Generalized Least Squares (PGLS). Mobbing propensity in our analyses was defined based on the frequency at which each species participated in the mobbing events during the experiments. A PGLS model incorporates information about phylogenetic signal in the covariance structure of residuals from the regression model (Symonds & Blomberg, 2014). We used the following global model to test the predictors of mobbing propensity (MobProp):

gls(MobProp ~ body_mass+abund_mob+guild+for_strata, correlation =corPagel(1, phy=phy, fixed=FALSE), method=“REML”).

We compared this global model with simpler models (removing insignificant predictors) to improve model fit. We removed foraging mode (which were not different from ~1) and recoded levels from categorical variables with less than 10 records in 108 bird species to avoid estimating wrong parameters with few replicates within certain unusual levels.

#### 2.5.6 Phylogenetic structure at the community scale

We tested whether the phylogenetic structure of the avian assemblage had a predictable effect on mobbing by using a Principal Coordinates of Phylogenetic Structure analysis (PCPS, Duarte et al., 2016). This analysis uses pairwise dissimilarities (based on phylogenetic patristic distances) between sampling points to explore clade distribution across meta‐communities weighted by species abundances. As an eigenvector analysis, PCPS produces orthogonal axes that can be further used in multivariate analyses, whereas the first PCPS axis with higher eigenvalues describe basal nodes contributing to phylogenetic structure, lower values can be interpreted as representing the structure of terminal nodes (Duarte et al., 2016).

Differences in phylogenetic and functional structure between sampling units (and their predictor variables, such as mobbing vs. census) were tested with a PERMANOVA. To avoid misinterpreting location and dispersion effects in comparisons of community structure, we used the PERMDISP method that evaluates the homogeneity of multivariate variances (Anderson & Walsh, 2013). The visual inspection with PCoA allows a full comprehension of differences in location and dispersion.

In addition, we controlled the effect of phylogenetic dependence in bird functional structure by using a recently proposed method, which decouples trait dissimilarity (which represents functional variation) from the phylogeny (de Bello, 2017). Then, we obtained a distance matrix (called dcFdist: decoupled trait dissimilarity) that was used in the same way we performed a PCPS with PERMANOVA (see above). Methods such as the PCPS is used for describing the spatial distribution of phylogenetic lineages across sites which, in turn, can be combined with distance‐based method to check whether some potential drivers (here, the mobbing behavior) can explain this phylogenetic structure at the community level (Duarte et al., 2016).

## 3 RESULTS

### 3.1 Avian surveys

The 115 playback experiments in the study area attracted a total of 2,052 individuals of 72 different species of birds of six Orders and 20 Families, representing ~70% of the species recorded in our censuses (Data available in the Dryad Digital Repository, see Data Accessibility for more information). Each playback experiment attracted an average of 5.79 (± 2.43) bird species and 8.26 (±3.81) individuals.

We found no statistical difference in the number of bird species (61) that were attracted to the experiments conducted in the early mornings (6.05 ± 2.44) and late afternoons (5.53 ± 2.41; *t = *1.628, *df* = 227.94, *p = *0.104). However, differences in the number of individuals were marginally significant. On average, more birds joined the mobs during the early morning (8.74 ± 3.61), than during the late afternoon (7.79 ± 3.96; *t = *1.911, *df* = 226.05, *p* = 0.057).

Eighty‐six percent of the species (62 out of 72) and 92% of the individuals (1,871 out of 2,052) attracted to the vocal stimulus of the Ferruginous Pygmy‐Owl were passerines (Order Passeriformes). Within the passerines, 26% (19) of the species and 47% of the individuals (904) attracted were flycatchers (Family Tyrannidae), followed by tanagers (Thraupidae), with 14% of the species and 13% of the individuals, antbirds (Thamnophilidae) with 11% of the species and 7% of the individuals, and members of the family Cardinalidae with 3% of species and 8% of the individuals. The most frequently recorded species in the experiments were the Mouse‐colored Tyrannulet *(Phaeomyias murina*), the Ultramarine Grosbeak (*Cyanoloxia brissonii*), and the Burnished‐buff Tanager (*Tangara cayana*; Table 1).

**Table 1:** The 20 most abundant and frequent species in the playback experiments conducted at the Fazenda Pau D’Arco. Abundance refers to the total number of individuals detected in the experiments, and frequency to the percentage of experiments where the species was detected

Species	Abundance	Frequency (%)
*Phaeomyias murina*	450	97.39
*Cyanoloxia brissonii*	158	69.57
*Tangara cayana*	140	58.26
*Euscarthmus meloryphus*	104	39.13
*Tyrannus melancholicus*	87	51.30
*Cantorchilus longirostris*	77	42.61
*Schistochlamys ruficapillus*	69	42.61
*Polioptila plumbea*	66	39.13
*Elaenia chilensis*	61	33.91
*Chlorostilbon lucidus*	46	27.83
*Thamnophilus pelzelni*	45	28.70
*Myiophobus fasciatus*	45	25.22
*Herpsilochmus sellowi*	42	28.70
*Coryphospingus pileatus*	39	22.61
*Myiopagis viridicata*	37	20.00
*Elaenia spectabilis*	34	23.48
*Hylophilus amaurocephalus*	31	20.00
*Neopelma pallescens*	27	19.13
*Camptostoma obsoletum*	27	20.00
*Empidonomus varius*	25	15.65

**Figure 2:**
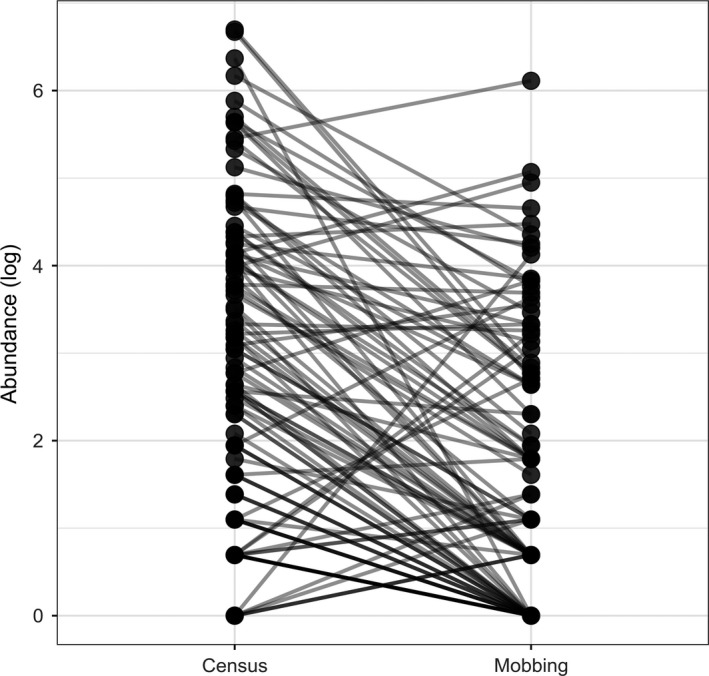
Cross‐correlation between species abundance and mobbing propensity. Each dot represents a different species, which is connected by a line to the same species; left column represents the individual log abundance observed in the censuses, whereas the right column represents the log of the mobbing propensity. This figure shows that the most abundant species found in the study area were not the most common species displaying mobbing behavior

We found that species abundance in the study area is not a good predictor of mobbing participation. The most abundant species recorded in the study area were not the most common species displaying mobbing behavior (Figure 2). There is a weak correlation between species abundance and mobbing propensity (*r* = 0.207, *p = *0.03).

### 3.2 Functional and phylogenetic structure of mobbing at the species and community levels

**Figure 3:**
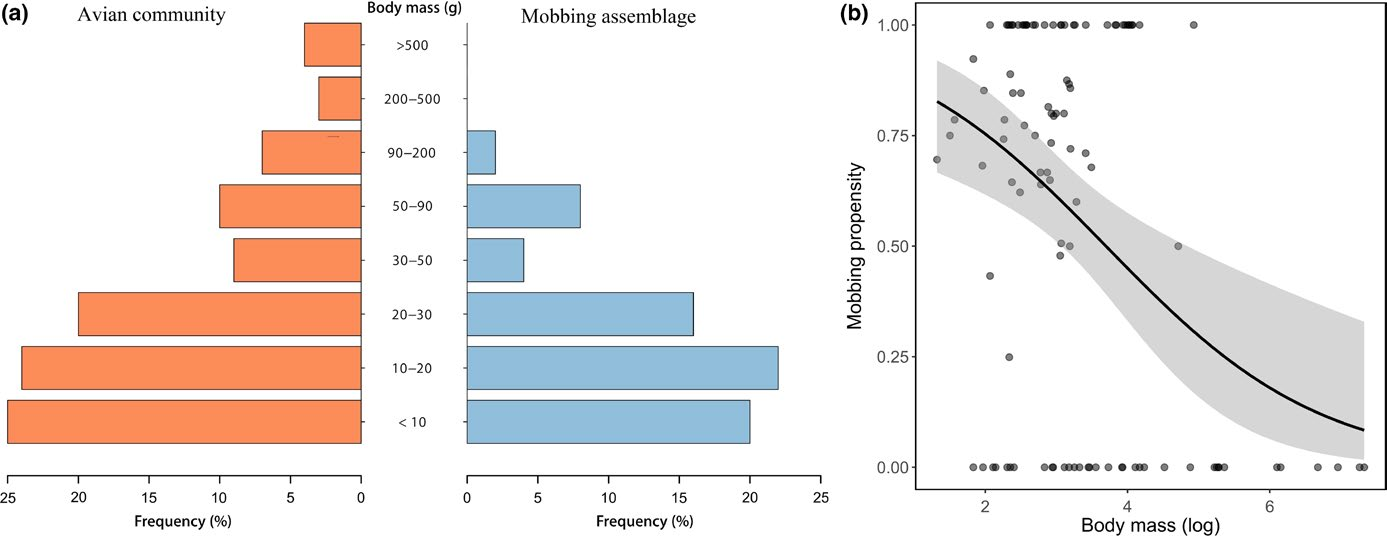
(a) Frequency of body mass classes in the entire avian community found at our study site (red) and in the mobbing assemblage (blue) (Data available in the Dryad Digital Repository, see Data Accessibility for more information). (b) Relationship between avian body mass and mobbing behavior; smaller birds are more likely to engage in the mobbing behavior

Body masses at the study area varied from 2.75 g in the Glittering‐bellied Emerald (*Chlorostilbon lucidus*) to 1,520 g in the Black Vulture (*Coragyps atratus*). The largest species attracted to the mobbing experiments was the Blond‐crested Woodpecker (*Celeus flavescens*), a medium‐sized (137.5 g) bird species (Data available in the Dryad Digital Repository, see Data Accessibility for more information). Comparing the assembly of mobbers with the avian community recorded during the censuses, we found that only smaller birds participated in the mobs (Figure 3a). In fact, body size appears as the best predictor for mobbing propensity (*F* = 6.21, *p* = 0.014); smaller birds are the most common in the mobs (Figure 3b).

All trophic guilds, except carnivores, were attracted to our mobbing experiments (Data available in the Dryad Digital Repository, see Data Accessibility for more information). More than half (53%) of the species (38) recorded in the experiments were insectivores, followed by insectivore/frugivores (28%, 20 species), and granivores (8%, 6 species), which together accounted for more than 90% of the species. In relation to foraging mode, almost half (46%) of the species (33) recorded during the playback experiments were substrate gleaners, followed by hunters (17%, 11 species), and fruits perchers (10%, 7 species; Data available in the Dryad Digital Repository, see Data Accessibility for more information). In relation to foraging strata, most birds recorded in the experiments were either canopy‐dwelling species (53%, 38 species), or understory birds (47%, 34 species). No open air or ground‐dwelling species were recorded in the mobbing experiments (Data available in the Dryad Digital Repository, see Data Accessibility for more information).

**Figure 4:**
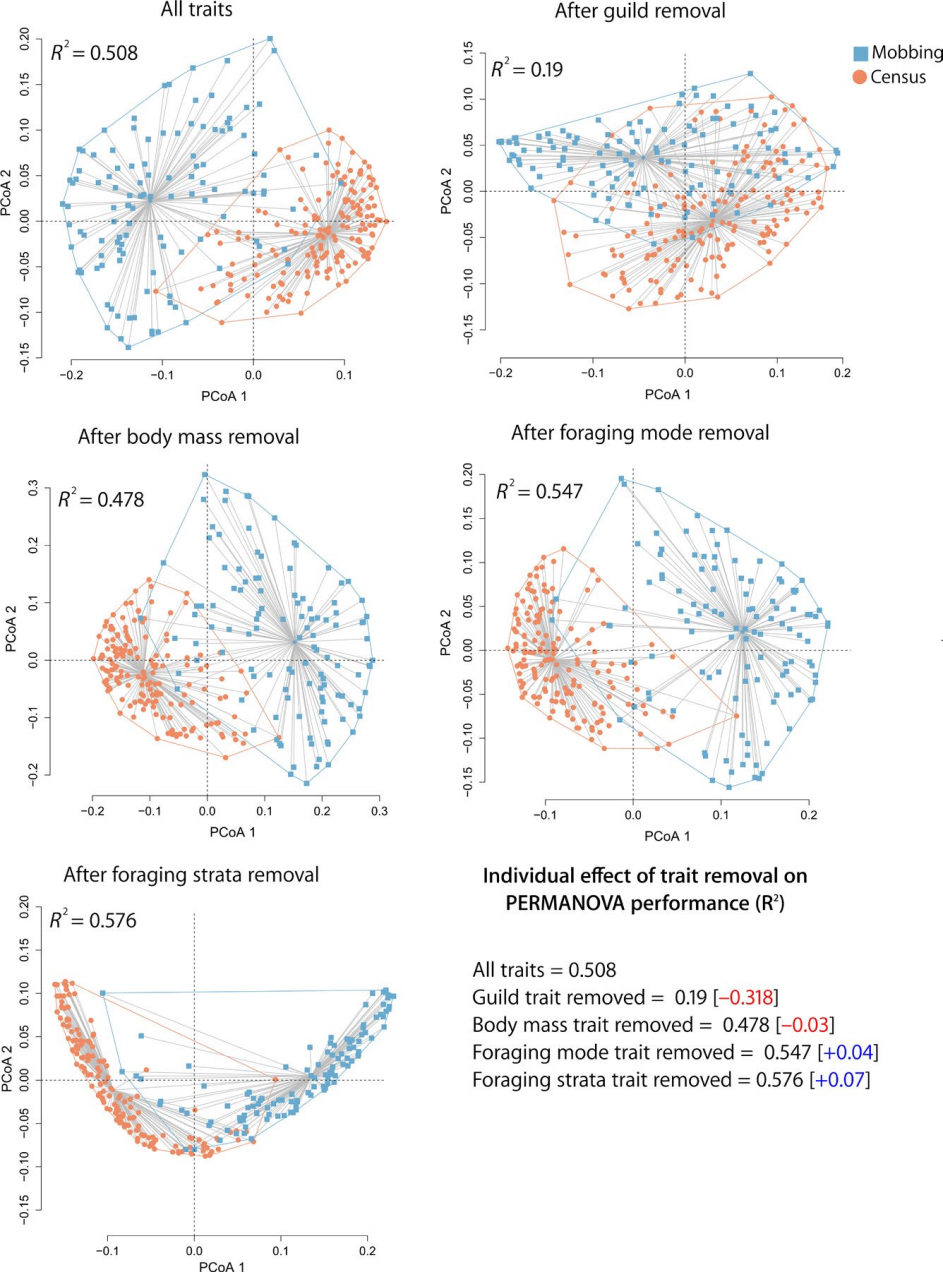
Principal Coordinates of Functional Structure Analysis including 115 mobbing experiments (in blue) and 162 community point counts (in orange) including all traits (global model) and partial models to identify the individual effects on Permanova’s performance after trait removal

At the species level, the best model explaining mobbing propensity included only body mass and guild (AICc = 124.75; Figure 4); body mass has a strong, negative effect on mobbing propensity (*F* = 9.63, *p = *0.003, Figure 4). Bird abundance and foraging strata were not retained as a plausible model (AICc = 143.85), which suggest they do not affect mobbing propensity. Species mobbing propensity has a significant, yet moderate phylogenetic signal (Pagel’s = λ0.40, *p < *0.006), which is very strong in relation to body mass (Pagel’s = λ0.99, *p < *0.001).

**Figure 5:**
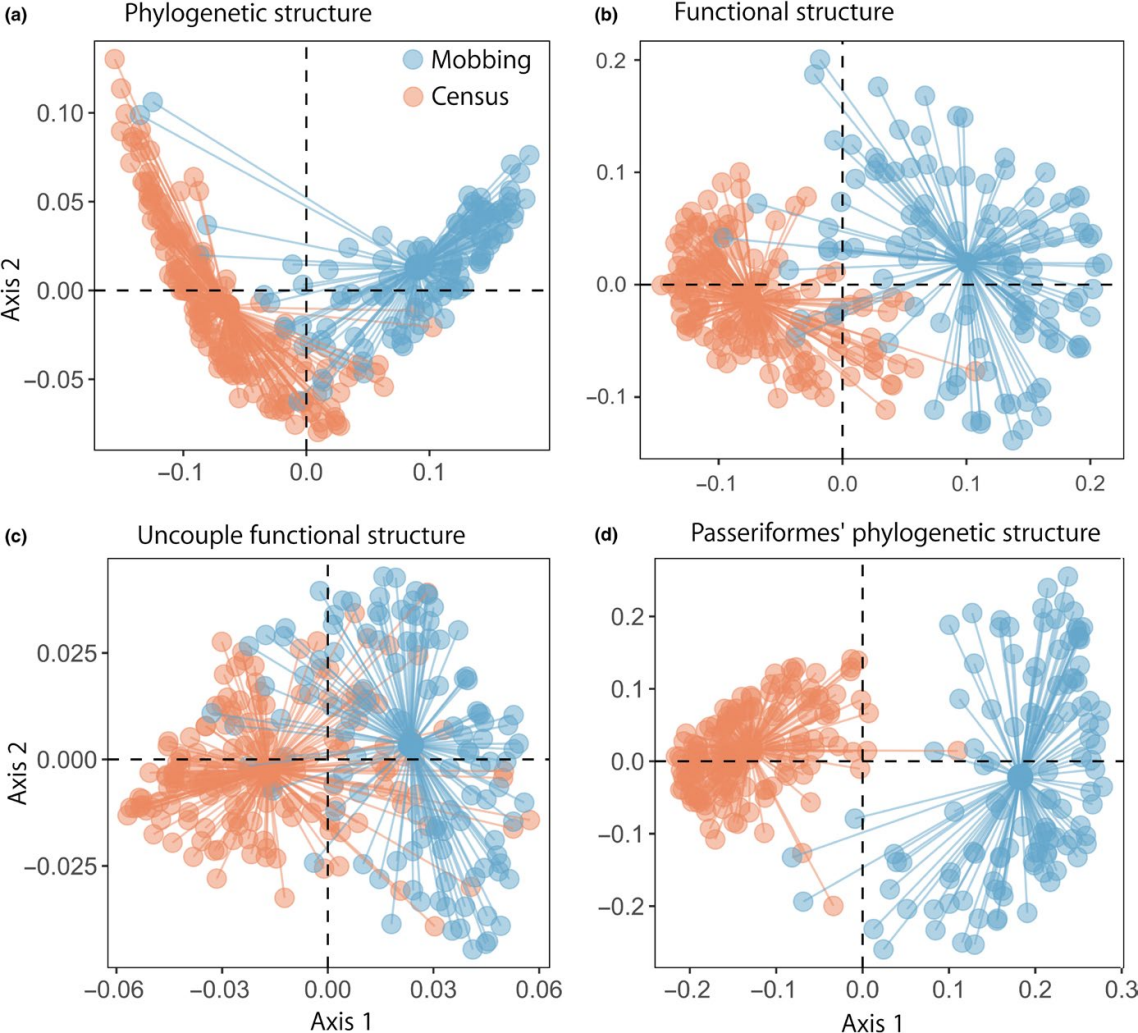
Principal Coordinates of Phylogenetic Structure Analysis including 115 mobbing experiments (in blue) and 162 community point counts (in orange) showing the patterns of phylogenetic and functional structure in mobbing behavior at the community scale

At the community level, we found that the phylogenetic structure of the mobbing assemblage is significantly different to that of the entire avian community (*R* ^2^ = 0.536, *p = *0.001, Figure 5a). This difference is strong throughout the first axis of the Principal Coordinates of Phylogenetic Structure and allows to predict which portion of the community engages in the mobbing behavior (Figure 5). Considering the traits analyzed, we found a strong difference in the functional structure between the bird assemblages resulting from the mobbing experiments and that obtained in our community‐wide avian surveys (*R* ^2^ = 0.508, *p = *0.001, Figures 4 and 5b). Both location and dispersion differ between the mobbing assemblage and the community detected during point counts (Figure 5). Confirming our previous analyses, global models also show guild and body mass as the main traits contributing to functional dissimilarity between the two groups, whereas foraging mode and strata had no significant effect on the functional structure of the assemblages (Figures 4 and 5b). Importantly, this functional structure is still evident even after controlling the effect of phylogenetic relatedness on functional traits (Figure 5c).

**Figure 6:**
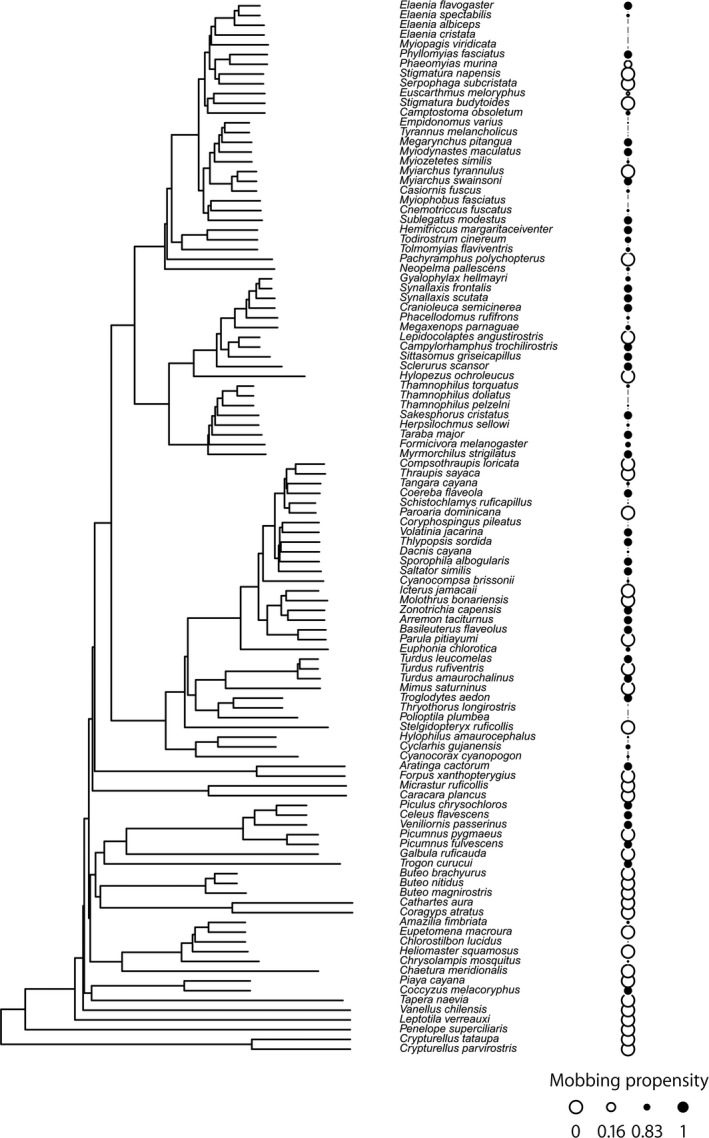
Mobbing propensity as a trait mapped in a phylogeny including all species recorded at the Fazenda Pau D’Arco. The colors of the dots refer to their propensity (black) or non‐propensity (white) of engaging in mobbing behavior. The size of the dot refers to this propensity (see Methods)

Despite the major presence of passerines in the mobs, we found a stronger phylogenetic structure that suggests that mobbing behavior is not a passerine‐exclusive trait (Figure 6). However, even within the Passeriformes (the most common order found in our mobbing experiments), there is a strong phylogenetic structure suggesting that the participation in the mobs represents a non‐random selection of closely related passerine families (*R* ^2^ = 0.619, *p = *0.001, Figure 5d). By mapping mobbing propensity in the community phylogeny, we found that even within the same clade, not all species participate in the mobs (Figure 6).

## 4 DISCUSSION

Our study offers a novel approach to investigate mobbing behavior by untangling which functional traits are more likely to predict which birds participate in the mobs. Furthermore, we demonstrated for the first time that although this behavior is widespread in the community, there is a significant phylogenetic relatedness in this anti‐predatory behavior. Hence, displaying mobbing behavior may benefit coexisting individuals from different species, and most importantly, it seems to be transmitted among close relatives. Three key conclusions emerge from this study: (a) the pervasiveness of the avian response to the vocalization of the Ferruginous Pygmy‐Owl in a tropical dry forest; (b) the predictive power of certain functional traits in explaining avian participation in the mobs; and (c) the phylogenetic relatedness of the subset of species that mob upon the owl.

### 4.1 Mobbing behavior in bird communities: the case of the Ferruginous Pygmy‐Owl

Mobbing on the Ferruginous Pygmy‐Owl is a widespread behavior throughout the Neotropics. The perused literature yielded a total of 247 bird species mobbing upon this owl throughout its range (Data available in the Dryad Digital Repository, see Data Accessibility for more information), representing nearly 6% of the entire Neotropical avifauna (Stotz, 1996). We have shown the pervasiveness of the avian response to the vocalization of the Ferruginous Pygmy‐Owl in a tropical dry forest of northeastern Brazil, where two‐thirds (66%) of the species recorded at our site responded to the podcasted voice of the predator. Despite representing one of the most well‐studied cases of mobbing behavior, half of the species recorded by us represent novel records of mobbing behavior against the Ferruginous Pygmy‐Owl. In addition, we added an entire order (Trogoniformes) and two families (Trogonidae and Scleruridae), previously unknown to mob on the Pygmy‐Owl.

The number of bird species responding to the vocalization of the Ferruginous Pygmy‐Owl may increase with further sampling. One of the leading hypotheses behind mobbing participation is the association of mobbing with offspring defense during the reproductive season (Doran et al., 2005; Ficken & Popp, 1996; Ostreiher, 2003). Therefore, it is possible that additional species participate in the mobs during the breeding season. Taken together, these results suggest mobbing behavior may be even more widespread in bird communities than previously thought.

Differently from other owl species, the Ferruginous Pygmy‐Owl is also active during daytime. Peak periods of Ferruginous Pygmy‐Owl prey delivery to their nest concentrate in the early mornings, middays, and evenings (Holt et al., 2017). This activity pattern may explain why we found as many species participating in our mobbing experiments during the early morning and late afternoons. On the other hand, we found a marginally significant effect on the period of the day in terms of the number of individuals detected during the experiments. The general higher activity of birds at dawn is likely responsible for the relatively higher participation in the experiments during the early mornings.

### 4.2 Phylogenetic and ecological predictors of mobbing behavior

A clear conclusion derived from this and previous studies is that although avian mobbing on raptorial birds is a widespread behavior, not all species respond equally to predator’s presence. While some species were present in virtually every mobbing experiment, others rarely showed up, independently of their general abundance in the area. For instance, the Mouse‐colored Tyrannulet *(Phaeomyias murina*) responded in 97% of the playback experiments. Although this species is relatively common in the study area, it was not included in the ten most frequent species, which suggests a strong association of this species with mobbing. In fact, we found a weak correlation between species abundance in our surveys and mobbing frequency, which emphasizes that bird abundance is not the only trait affecting mobbing propensity. Here, we demonstrated that including functional and phylogenetic components increases our predictive power about which species engage in this aggressive behavior. This approach is independent of scale and taxonomy and should improve future studies with other mobbing organisms.

At the species level, the two traits that best predict species participation in the mobs are body mass and foraging guild; our explanatory power decreased significantly when these traits are excluded from the analyses. Smaller species tend to engage more in mobbing than larger ones, possibly because they represent those species more likely to be predated upon. In fact, the most frequent species engaging in owl mobbing are those most often eaten by them (Gehlbach & Leverett, 1995). The predominance of smaller individuals (97% of the mobbing species were birds smaller than 100 g) could be explained by the fact that small birds are more commonly attacked by Ferruginous Pygmy‐Owls. Body mass is one of the most important functional traits determining prey vulnerability to predation risk (Hua et al., 2016). It may define whether a species can be caught, subdued, and consumed by a predator (Hua et al., 2016). Potential preys are known to adjust the strength of their mobbing behavior according to their risk of predation (Motta‐Junior & Santos‐Filho, 2012; Sandoval & Wilson, 2012; Tilgar & Moks, 2015). Therefore, mobbing may vary in response to predation pressure (Dutour, Lena, & Lengagne, 2016; Sandoval & Wilson, 2012). In fact, mobbing intensity by potential preys has been associated with their prevalence in the diet of the Eurasian Pygmy‐Owl (*Glaucidium passerinum*); the more a species was preyed upon, more likely it was to exhibit a mobbing response (Dutour et al., 2016). Passerine birds represented 40% of the diet of the Ferruginous Pygmy‐Owl in northern Bahia (Lima & Lima‐Neto, 2008) and represented the most common prey item in the diet of the same species in the dry Chaco of Argentina (Carrera, Fernández, Kacoliris, Pagano, & Berkunsky, 2008), and hummingbirds also were documented to represent one of the top main feeding items in southwestern Brazil (Sazima, 2015). Unfortunately no data are available from the Brazilian Caatinga and future studies on the Ferruginous Pygmy‐Owl’s diet will enlighten the association between food preference, predation risk, and the propensity of mobbing behavior. That larger birds do not participate in the mobs supports the selfish behavior hypothesis (e.g., Ostreiher, 2003), as birds would only participate if they may be predated upon, and weakens the parental care hypothesis (e.g., Ostreiher, 2003), as even larger birds have smaller offspring.

Feeding guild also represented an important trait to predict species participation in the mobs. Insectivores, insectivores/frugivores, and granivores represented ~90% of all species displaying mobbing behavior (Data available in the Dryad Digital Repository, see Data Accessibility for more information). It remains unclear to us how exactly the guild may influence the mobbing behavior other than representing their activity patterns while looking for food, but foraging mode and foraging strata did not represent meaningful traits in our models.

At the community level, we found significant differences between the phylogenetic structure of the mobbing assemblage compared to that of the entire avian community, suggesting that phylogenetic relatedness is an important variable to explain mobbing behavior. Passerines represented almost 90% of the mobbers, and among them tyrant flycatchers, antbirds, and tanagers, were most represented in the mobs, whereas this would weaken Altman’s (1956) hypothesis that presence or absence of some species in the mobs could not be correlated with species’ taxonomic position, we found that some clades within the passerines are formed both by species that participate in mobbing behavior and by species that do not participate. Indeed, even within passerines, there are some clades that do not engage in mobbing behavior, which reinforces that phylogenetic relatedness interacts with ecological predictors to affect this behavior.

Based in our results, we argue that there is a predictable difference between the phylogenetic and the functional structure of mobbers compared to the entire avian community. The implication of these results is two‐folded; first, it emphasizes that mobbing behavior, although widespread, could be phylogenetically conserved (e.g., Randler, 2012), but that ecological predictors (such as guild) may also help explain this anti‐predatory behavior.

## 5 CONCLUSIONS

Our results indicate that the mobbing behavior is widespread in the avian community studied, and by being shared by different avian orders it probably appeared early in the evolutionary history of birds. The maintenance of the mobbing behavior through evolutionary time seems to be transmitted among close relatives, especially within the passerines. However, whether this behavior originated within this lineage and spread to other smaller non‐passerine birds, or simply developed within preyed species remains to be clarified. Our results also provide empirical evidence that guild and body mass are essential traits explaining the propensity of bird species to participate in mobbing behavior. Accordingly, we argue that two key insights can stimulate studies in other systems and organisms: on the one hand, prey species sharing similar food sources (same guild) may facilitate encounter with predators which, in turn, may favor the development of defensive behaviors. On the other hand, small‐bodied organisms tend to respond more aggressively to the presence of a natural enemy. Data on the Owl’s diet and the social networks (i.e., order of mobbing attendance) related to mobbing behavior are two central topics that will allow us advance on this elaborated animal behavior.

## CONFLICT OF INTEREST

The authors have no conflict of interests.

## AUTHORS CONTRIBUTIONS

HSL, FMGL‐C, and LNN designed the study; HSL, FMGL‐C, and JRR collected field data; HSL, LNN, and TG‐S analyzed the data; HSL wrote the manuscript with guidance from LNN and TG‐S; all the authors edited and revised the manuscript.

## DATA ACCESSIBILITY

All data used in this publication, including bird species who display mobbing behavior against the Ferruginous Pygmy‐Owl (*Glaucidium brasilianum*) in the Neotropics and the functional traits data for species registered in the study area and in the mobbing behavior, are archived and available in the Dryad Digital Repository: https://doi.org/10.5061/dryad.4bp16g2.
